# Draft Genome Sequences of *Sanguibacteroides justesenii*, gen. nov., sp. nov., Strains OUH 308042^T^ (= ATCC BAA-2681^T^) and OUH 334697 (= ATCC BAA-2682), Isolated from Blood Cultures from Two Different Patients

**DOI:** 10.1128/genomeA.00005-15

**Published:** 2015-03-26

**Authors:** Thomas Vognbjerg Sydenham, Henrik Hasman, Ulrik Stenz Justesen

**Affiliations:** a Department of Clinical Microbiology, Odense University Hospital, Odense, Denmark; b The Microbial Genomics and Antimicrobial Resistance Group, National Food Institute, Technical University of Denmark, Lyngby, Denmark

## Abstract

We announce here the draft genome sequences of *Sanguibacteroides justesenii*, gen. nov., sp. nov., strains OUH 308042^T^ (= DSM 28342^T^ = ATCC BAA-2681^T^) and OUH 334697 (= DSM 28341 = ATCC BAA-2682), isolated from blood cultures from two different patients and composed of 51 and 39 contigs for totals of 3,385,516 and 3,410,672 bp, respectively.

## GENOME ANNOUNCEMENT

The introduction of 16S rRNA gene sequencing for possible routine use in clinical microbiology has led to increased species differentiation for anaerobic bacteria that were previously erroneously identified or only partially identified by traditional biochemical methods (1). From blood cultures from two different patients, we isolated Sanguibacteroides justesenii, gen. nov., sp nov., with the closest 16S rRNA gene sequence match of 90.5% similarity to Butyricimonas virosa (T.V.S. and U.S.J., unpublished data).

Here, we announce the availability of the draft genome sequences of the two clinical isolates, one being the type strain for the newly defined *Sanguibacteroides* genus.

Genomic DNA was purified using the MasterPure DNA purification kit (Epicentre Biotechnologies, Madison, WI, USA), according to the manufacturer’s instructions. The libraries were prepared using the Nextera XT kit (Illumina, Essex, United Kingdom), and sequencing was performed on the Illumina MiSeq platform with a 2 × 250 paired-end run, with adapter trimming performed by the MiSeq Reporter software, resulting in 656,492 and 1,192,570 paired reads and a total of 328 and 596 meganucleotides for OUH 308042T and OUH 334696, respectively.

The reads were assembled *de novo* using the SPAdes 3.0.0 assembler implemented on the Orione Galaxy Web server (http://orione.crs4.it/), with K values equal to 21, 33, 55, 77, 99, and 127, as suggested in the SPAdes 3.0.0 manual, and contigs of <500 bp in length were removed (2, 3). Following submission to the NCBI whole-genome shotgun submission portal, contigs tagged as contaminations by the contamination screen performed by NCBI staff were removed, and the contigs were trimmed of sequences tagged as adapter sequences. Assembly metrics were calculated using the Quast Web server (http://quast.bioinf.spbau.ru/) (4). For strain OUH 308042^T^, this genome assembly resulted in a total draft genome length of 3,385,516 bp, made up of 51 contigs with a contig *N*_50_ of 469,346 and a G+C content of 42.31%. For strain OUH 334697, the genome assembly resulted in a total draft genome length of 3,410,672 bp, consisting of 39 contigs with a contig *N*_50_ of 483,020 and a G+C content of 42.02%. The genomes were annotated by the NCBI Prokaryote Genome Annotation Pipeline (PGAP) version 2.0 (5), obtaining 2,678 and 2,653 protein-coding genes for OUH 308042^T^ and OUH 334697, respectively.

OUH 308042^T^ was blasted against the MvirDB virulence database (http://mvirdb.llnl.gov/) (6), revealing no significant hits to virulence genes in that database. PGAP annotation resulted in five predicted β-lactamases in both strains corresponding to the penicillin resistance observed for the strains (T.V.S. and U.S.J., unpublished data).

The availability of these two draft genome sequences will enable more in-depth studies on the virulence mechanisms and antibiotic resistance properties of this opportunistic pathogen.

### Nucleotide sequence accession numbers.

These whole-genome shotgun projects have been deposited in DDBJ/EMBL/GenBank under the accession numbers JPIU00000000 and JPIT00000000 for strains OUH 308042^T^ and OUH 334697, respectively. The versions described in this paper are the first versions, JPIU01000000 and JPIT01000000, respectively.
